# Timing of general anesthesia for pediatric patients recovering from COVID-19: a prospective cohort study

**DOI:** 10.1186/s12871-023-02390-9

**Published:** 2024-01-02

**Authors:** Dinghuan Zhao, Wei Liu, Zhao Zhang, Yuting Li, Jun Luo, Weiqiang Zheng, Ruiqiang Sun

**Affiliations:** Department of Anesthesiology, Tianjin Eye Hospital, No. 4 Gansu Road, Heping District, Tianjin, 300022 China

**Keywords:** Child, COVID-19, Anesthesia, General, Laryngeal masks

## Abstract

**Objective:**

To explore the timing of general anesthesia for pediatric patients who have recovered from novel coronavirus infection and summarize anesthesia-related complications.

**Methods:**

We summarized the perioperative management of children under 14 years of age who underwent general anesthesia in our hospital according to national epidemic prevention and control requirements. We compared the incidence of postoperative pulmonary complications within 2 weeks (Group A), 3–4 weeks (Group B), and 5–6 weeks (Group C) after COVID-19 recovery.

**Results:**

There were differences among the three groups in terms of decreased blood oxygen saturation (< 94%), secretions, and coughing during the PACU period. The risk of low blood oxygen saturation during PACU decreased as the time of COVID-19 recovery extended in the three groups. Compared to Group A, the risk of low blood oxygen saturation was lower in Group B. The presence of respiratory symptoms and a body temperature above 40℃ increased the risk of decreased blood oxygen saturation. The proportion of children aged 11–14 years and children with high fever experiencing decreased blood oxygen saturation during PACU was higher in Groups A and B. Among the three groups, children with respiratory symptoms and longer illness duration had a higher proportion of decreased blood oxygen saturation during PACU.

**Conclusion:**

Pediatric patients who have recovered from COVID-19 for more than 2 weeks have a lower risk of postoperative complications after general anesthesia. For children with respiratory system symptoms or high fever, there is a higher risk of transient blood oxygen saturation decrease during PACU. For older children, those with high fever, respiratory system symptoms, or longer illness duration, it is recommended to appropriately extend the time from COVID-19 recovery to surgery.

## Introduction

The COVID-19 pandemic has posed significant challenges to perioperative management. Due to the associated risks of anesthesia complications and exposure for perioperative staff and patients, many elective surgeries have been postponed or canceled. Compared to adults, a higher proportion of pediatric patients infected with the novel coronavirus exhibit asymptomatic infections, and their infectivity is significantly lower than that of adult patients [1]. Omicron rapidly replaced Delta as the dominant variant worldwide since November/December 2021 [2]. Omicron has shown significantly reduced pathogenicity compared to previous strains [3], with 90% of infected children experiencing mild symptoms [4]. The main symptoms include upper respiratory tract infections, fever, sore throat, cough, and wheezing [5, 6]. 3–25% of pediatric patients may experience long-term symptoms after COVID-19, primarily characterized by fatigue, emotional disturbances, and sleep disorders [7]. Previous studies suggested that asymptomatic pediatric patients who have recovered from COVID-19 for 28 days have reduced risks of virus transmission and anesthesia-related complications [8], recommending that these patients undergo general anesthesia surgery four weeks after recovery [9]. However, due to the lower pathogenicity of Omicron, the anesthesia risks for children who have been infected with Omicron are also reduced. Some studies suggest that pediatric patients who have been infected with Omicron can undergo surgery as early as 10 days after infection without an increased incidence of postoperative adverse reactions [10]. However, there is currently no definitive consensus on the timing of general anesthesia surgery for pediatric patients who have been infected with Omicron.

This study is an observational clinical study that analyzes data from pediatric patients who underwent general anesthesia with a laryngeal mask while retaining spontaneous respiration in our hospital from February 7, 2023, to May 20, 2023. The study aims to compare the impact of different timing of surgery after COVID-19 recovery on the incidence of perioperative complications in pediatric patients and explore the timing selection for surgery in pediatric patients after COVID-19 recovery.

## Materials and methods

### General information

This study is a single-center prospective cohort study approved by the Medical Ethics Committee of Tianjin Eye Hospital (KY-2,023,010). The inclusion criteria were as follows: (1) age ≤ 14 years old; (2) positive COVID-19 antigen test within 6 weeks before surgery; (3) undergoing general anesthesia with a laryngeal mask while retaining spontaneous respiration; (4) ASA classification I-II; (5) no fever or upper respiratory symptoms before surgery; (6) completion of a 7-day follow-up. The exclusion criteria were as follows: (1) severe congenital diseases or oropharyngeal deformities; (2) preoperative fingertip oxygen saturation < 95%; (3) intraoperative mechanical ventilation or endotracheal intubation; (4) incomplete follow-up.

Based on the above criteria, a total of 317 pediatric cases were included from February 7, 2023, to May 20, 2023, including 259 cases of strabismus correction, 41 cases of orbital and ocular plastic surgery, 8 cases of ocular trauma, 5 cases of posterior segment surgery, 2 cases of corneal surgery, and 2 cases of enucleation. All patients received intramuscular injection of atropine 0.01–0.02 mg/kg (< 0.8 mg) 30 min before surgery. Anesthesia was induced and maintained using sevoflurane-remifentanil-propofol general anesthesia, with the laryngeal mask retaining spontaneous respiration. Intraoperative monitoring included SpO_2_ (oxyhemoglobin saturation), ECG (electrocardiograph), HR (heart rate), BP (blood pressure), BIS (bispectral index), temperature, and respiratory parameters. After surgery, intravenous injection of ondansetron 0.2 mg/kg (< 4 mg) was given, and the patient was transferred to the PACU (post-anesthesia care unit) with the laryngeal mask. The laryngeal mask was removed after the patient regained consciousness, and if the Steward score was greater than 4, the patient was transferred back to the ward.

### Grouping criteria

The included cases were all pediatric patients who tested positive for COVID-19 within 6 weeks before surgery (confirmed by medical institution testing or self-check at home). The recovery criteria for COVID-19 infection were as follows: normal body temperature for more than 24 h, significant improvement in pre-existing symptoms (including respiratory symptoms, diarrhea, vomiting, and other symptoms), and stable vital signs. Based on the time from COVID-19 recovery to surgery, the patients were divided into three groups: Group A: surgery within 2 weeks after recovery; Group B: surgery 3–4 weeks after recovery; Group C: surgery 5–6 weeks after recovery.

### Observational indicators

Baseline indicators of patients were recorded, including gender, age and BMI. Record the severity of the disease in children infected with the novel coronavirus, including the presence of respiratory symptoms, disease duration, highest body temperature, and treatment.

The primary observed outcome indicator in this study is the transient decrease in peripheral capillary SpO_2_ to < 94% during the post-anesthesia care unit (PACU) period while breathing ambient air. Other outcome indicators include coughing, increased secretions, sore throat, restlessness and laryngospasm during PACU and fever, antibiotic treatment, emergency department visits, and respiratory support during the postoperative period. The PACU physician is responsible for observing and recording the outcome indicators during the PACU period. The anesthesiologist, PACU physician, data follow-up personnel, and data statistician are all blinded to the group assignments. All study participants receive standardized training before the start of the study.

### Statistical methods

Statistical analysis is performed using IBM SPSS 22.0 software. Normally distributed data with homogeneous variances are presented as mean ± standard deviation, and one-way analysis of variance (ANOVA) is used for comparisons among the three groups. Pearson’s chi-square test is used for comparisons of categorical data. When the conditions for Pearson’s test are not met, Fisher’s exact test is used for the chi-square test. Bonferroni correction was used for multiple-comparison correction. Multiple logistic regression analysis is used to assess the risk factors that may contribute to decreased oxygen saturation. *P* value less than 0.05 (*P* < 0.05) is considered statistically significant.

## Results

There were no statistically significant differences among the three groups in terms of age, gender, BMI, respiratory symptoms during infection, and maximum body temperature (MBT). There was a statistical difference in the treatment comparison due to two patients in Group B requiring hospitalization, which caused a difference in the comparison among the three groups. However, upon investigation, it was found that the hospitalization reasons for these patients were unrelated to COVID-19. One patient was hospitalized for retinoblastoma during the infection period, while the other patient was hospitalized for trauma during the infection period (See Table 1).

**Table 1:** Demographics

Group and procedure characteristics	A	B	C	F/χ^2^	*P*
Total	70	123	124	-	-
Gender (n, %)					
Male	34 (48.6)	63 (51.2)	64 (51.6)	0.181	0.927
Female	36 (51.4)	60 (48.8)	60 (48.4)		
Age (y)	7.77 ± 3.051	8.72 ± 3.121	9.27 ± 2.846	5.551	0.004
BMI > 28 (n, %)	2 (2.9)	12 (9.8)	6 (4.8)	4.337	0.102
Respiratory symptoms (n, %)	27	63	61	0.022	0.085
MBT (n, %)					
< 38℃	12 (17.1)	18 (14.6)	20 (16.1)	2.195	0.706
38-40℃	51 (72.9)	97 (78.9)	90 (72.6)		
> 40℃	7 (10.0)	8 (6.5)	14 (11.3)		
Disease course (n, %)					
< 3 days	43 (61.4)	64 (52.0)	79 (63.7)	4.366	0.362
3-7 days	17 (24.3)	37 (30.1)	25 (20.2)		
> 7 days	10 (14.3)	22 (17.9)	20 (16.1)		
Treatment (n, %)					
Housebound	64 (91.4)	119 (96.7)	115 (92.7)	8.276	0.042
Emergency visit	6 (8.6)	2 (1.6)	9 (7.3)		
Hospital admission	0	2 (1.6)	0		

There were differences among the three groups in terms of transient blood oxygen desaturation (< 94%), secretions, and coughing during the PACU period (*P* < 0.05). As the time of COVID-19 recovery extended, the incidence of postoperative respiratory complications in the PACU decreased. The transient blood oxygen desaturation rates were 32.9% vs. 13.8% vs. 4.8% in the three groups, coughing rates were 37.1% vs. 28.5% vs. 18.5%, and secretion rates were 51.4% vs. 22% vs. 10.5%. All of the respiratory complications mentioned above were appropriately managed, and no serious complications occurred. There was no statistically significant difference among the three groups in terms of spasm restlessness and throat pain. There were no statistically significant differences in respiratory complications at 24 h and 7 days postoperatively (See Table 2).

**Table 2:** Postoperative complications classification and comparison among the three groups

Group and procedure characteristics		A	B	C	χ^2^	*P*
PACU period (n, %)	SpO_2_ < 94%	23 (32.9)^*§^	17 (13.8)^*#^	6 (4.8)^#§^	28.391	< 0.001
	Coughing	26 (37.1)^§^	35 (28.5)	23 (18.5)^§^	8.338	0.015
	Secretions	36 (51.4)^*§^	27 (22.0)^*#^	13 (10.5)^#§^	41.604	< 0.001
	Sore throat	41 (58.6)	54 (43.9)	51 (41.1)	5.854	0.054
	Restlessness	18 (25.7)	24 (19.5)	22 (17.7)	1.822	0.402
	Laryngospasm	5 (7.1)^*^	1 (0.8)^*^	2 (1.6)	6.246	0.044
24 h postoperatively (n, %)	Fever	17 (24.3)	22 (17.9)	23 (18.5)	1.293	0.524
	Antibiotic treatment	3 (4.3)	4 (3.3)	4 (3.2)	0.363	0.856
	Emergency visit	0	0	2 (1.6)	2.111	0.350
	Respiratory support	0	0	0	-	-
7 days postoperatively (n, %)	Fever	4 (5.7)	5 (4.1)	5 (4.0)	0.512	0.830
	Antibiotic treatment	1 (1.4)	4 (3.3)	1 (0.8)	1.847	0.390
	Emergency visit	3 (4.3)	3 (2.4)	1 (0.8)	2.612	0.264
	Respiratory support	0	0	0	-	-

^*^P < 0.05 in Group A vs Group B

^#^*P* < 0.05 in Group B vs Group C

^§^*P* < 0.05 in Group A vs Group C

With the extension of the recovery time after infection, the risk of transient blood oxygen reduction in the PACU decreased (*P* < 0.05). Compared to Group A, the risk of transient blood oxygen reduction was lower in Group B (OR: 0.150; 95% CI [0.058, 0.389]; *P* < 0.001) and Group C (OR: 0.038; 95% CI [0.011, 0.126]; *P* < 0.001). The age of the children, the duration of illness, and treatment measures had no effect on transient blood oxygen reduction in the PACU. Having respiratory symptoms during COVID-19 infection (OR: 6.911; 95% CI [1.575, 30.325]; *P* = 0.010) and having a body temperature above 40℃ during infection (OR: 10.565; 95% CI [2.004, 55.713]; *P* = 0.005) increased the risk of transient blood oxygen reduction in the PACU (See Table 3).

**Table 3:** Multivariate logistic regression results for PACU transient decrease in oxygen saturation

Variable	SpO_2_ < 94% (n, %)	Odds ratio	95% CI	*P*
Group				
A	23 (32.9)	1 [Reference]	-	-
B	17 (13.8)	0.150	0.058, 0.389	< 0.001
C	6 (4.8)	0.038	0.011, 0.126	< 0.001
Age				
0–5	6 (12)	1 [Reference]	-	-
6–10	18 (11.1)	1.154	0.320, 4.157	0.827
11–14	22 (21)	1.832	0.486, 6.907	0.371
Temperature				
< 38℃	4 (8.0)	1 [Reference]	-	-
38–40℃	30 (12.6)	1.585	0.439, 5.720	0.482
> 40℃	12 (41.4)	10.565	2.004, 55.713	0.005
Respiratory symptom				
Yes	40 (24.8)	6.911	1.575, 30.325	0.010
No	6 (3.8)			
Disease course				
< 3 days	9 (4.8)	1 [Reference]	-	-
3–7 days	18 (22.8)	1.591	0.419, 6.048	0.496
> 7 days	19 (36.5)	2.801	0.704, 11.147	0.144
Treatment				
Housebound	41 (13.8)	1 [Reference]	-	-
Emergency visit	5 (29.4)	0.403	0.069, 2.372	0.236
Hospital admission	0	0	0	1.000

In Group A and Group B, older children aged 11–14 (*P* = 0.009; *P* = 0.011) and children with high fever (*P* = 0.007; *P* = 0.005) had a higher proportion of transient blood oxygen reduction during the PACU period. Among the three groups of children, those with respiratory symptoms (A/B: *P* < 0.001; C: *P* = 0.035) and longer duration of illness (A/B: *P* < 0.001; C: *P* = 0.027) had a higher proportion of transient blood oxygen reduction during the PACU period (See Table 4).

**Table 4:** Subgroup analysis of PACU transient decrease in oxygen saturation

Subgroup and procedure characteristics		Group A			Group B			Group C		
		SpO_2_ < 94%	χ^2^	*P*	SpO_2_ < 94%	χ^2^	*P*	SpO_2_ < 94%	χ^2^	*P*
Age (n, %)	0-5	4 (16.7)	9.335	0.009	2 (11.1)	9.016	0.011	0	1.508	0.600
	6-10	9 (30.0)			4 (6.3)			5 (7.4)		
	11-14	10 (62.5)			11 (26.8)			1 (2.1)		
Temperature (n, %)	< 38℃	4 (33.3)	9.213	0.007	0	9.481	0.005	0	3.076	0.187
	38-40℃	13 (25.5)			13 (13.4)			4 (4.4)		
	> 40℃	6 (85.7)			4 (50.0)			2 (14.3)		
Respiratory symptom (n, %)	Yes	18 (62.1)	19.151	< 0.001	16 (25.8)	15.077	< 0.001	6 (8.6)	4.864	0.035
	No	5 (12.2)			1 (1.6)			0		
Disease course (n, %)	< 3 days	5 (11.6)	24.054	< 0.001	3 (4.7)	18.038	< 0.001	1 (1.3)	6.442	0.027
	3–7 days	10 (58.8)			5 (13.5)			3 (12.0)		
	> 7 days	8 (80.0)			9 (40.9)			2 (10.0)		
Treatment (n, %)	Housebound	19 (29.7)	3.400	0.065	16 (13.4)	2.649	0.453	6 (5.2)	0.493	1.000
	Emergency visit	4 (66.7)			1 (50.0)			0		
	Hospital admission	0			0			0		

**Fig. 1:**
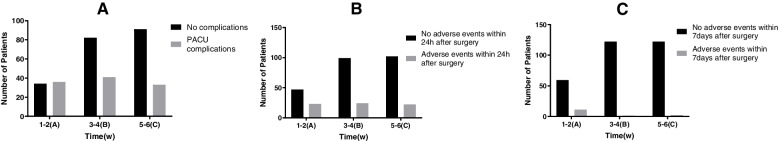
Complications after surgery

As shown in Fig. 1, with the prolonged recovery time of children after COVID-19, the incidence of anesthesia-related complications during the PACU period, 24 h after surgery, and 7 days after surgery decreased.

## Discussion

The results of this study showed that with the prolonged recovery time after COVID-19, the incidence of transient blood oxygen reduction, coughing, and increased secretions in children during the PACU period significantly decreased. The postoperative complications during the PACU period, 24 h after surgery, and 7 days after surgery also significantly decreased in children. Compared to undergoing surgery within 2 weeks after COVID-19 recovery, children who underwent surgery 3–6 weeks after recovery had a significantly lower risk of transient blood oxygen reduction during the PACU period. However, children with high fever (> 40℃) or significant respiratory symptoms during COVID-19 infection had a higher risk of transient blood oxygen reduction during the PACU period. Subgroup analysis results showed that among the three groups of children, those with respiratory symptoms or a duration of illness exceeding 7 days during COVID-19 infection had a higher proportion of transient blood oxygen reduction during the PACU period. For children who received general anesthesia within four weeks after infection (Group A and Group B), older children aged 11–14 or children with high fever (> 40℃) during COVID-19 infection had a higher proportion of transient blood oxygen reduction during the PACU period.

The laryngeal mask airway (LMA) is currently extensively utilized as a device for airway management in pediatric anesthesia, offering notable advantages such as ease of operation, reduced stimulation and injury risks, and excellent patient tolerance. Airway reactivity exhibits age-related variations, with children’s airways being more susceptible to irritation compared to adults [11]. In contrast to tracheal intubation, the laryngeal mask demonstrates reduced perioperative complications including lower rates of coughing, postoperative sore throat, and postoperative nausea and vomiting [12]. The incidence of postoperative complications such as bronchospasm, laryngospasm, and soft tissue injury is significantly higher after endotracheal intubation as compared to LMA insertion [13]. Although the utilization of general anesthesia with a laryngeal mask can effectively mitigate the risk of postoperative cough in pediatric patients afflicted with upper respiratory tract infections (URTI) [14], it is noteworthy that children who have recently experienced URTI possess a higher susceptibility to airway-related complications following general anesthesia, as compared to those with healthy airways [15]. In order to minimize the potential occurrence of airway-related complications during their stay in PACU, this study implemented a protocol for removing the laryngeal mask while ensuring that the child remained awake [16]. The findings derived from this investigation indicate that there exists a decline in the incidence of airway-related complications during children’s PACU stay as the duration between COVID-19 recovery and surgery increases.

The cases included in this study were all children who underwent general anesthesia surgery in our hospital from February 7, 2023, to May 20, 2023. All of these patients had been infected with the novel coronavirus within six weeks before the surgery. According to epidemiological studies on COVID-19 [2], Omicron became the predominant variant globally starting from January 2022. Therefore, it can be inferred that the infected strains in the pediatric COVID-19 rehabilitation patients included in this study were almost exclusively Omicron variants. Compared to previous strains of the novel coronavirus, Omicron variants have significantly reduced virulence [17, 18] and predominantly cause upper respiratory tract symptoms with a lower incidence of pneumonia [5]. One possible reason is that Omicron variants alleviate COVID-19-related symptoms through CD8^+^ T cell amplification and cross-reactivity with memory T cells [19]. Children have a more diverse primary antibody response to Omicron variants due to their relatively primitive immune system, resulting in a stronger and broader immune response against the novel coronavirus [20]. Additionally, the lower expression of key host proteins (ACE2, TMPRSS2, furin) for the SARS-CoV-2 virus in children may explain the milder symptoms observed in this age group [21]. Considering that there are more mild cases and faster recovery in children infected with Omicron, this study aims to explore the timing of anesthesia surgery after their rehabilitation and summarize the associated complications.

Previous studies have shown that the incidence of pulmonary complications after general anesthesia in children with URTI ranges from 24–30% [9, 22]. For children who still exhibit significant active symptoms of COVID-19 infection, general anesthesia should be avoided [9, 23]. For previous strains of the virus, it is recommended that children with mild symptoms undergo surgery at least four weeks after recovery [9]. The results of this study indicate that the risk of postoperative complications is lower when pediatric patients who have recovered from Omicron infection undergo surgery at least two weeks after rehabilitation. However, for children with significant respiratory symptoms during the COVID-19 infection period, the risk of transient blood oxygen desaturation during PACU period increases significantly. This finding is consistent across subgroup analyses. Therefore, it is advisable to further delay the timing of general anesthesia surgery after COVID-19 rehabilitation for children with severe respiratory symptoms during the infection period.

Long COVID refers to the persistent, newly emerging, or recurrent health issues experienced by patients after contracting COVID-19 [24]. According to the NICE guidelines, long COVID symptoms include symptoms that persist for 4–12 weeks after COVID-19 infection and those that appear after 12 weeks [25]. These symptoms mainly involve respiratory and neurological manifestations, such as cough, fatigue, and weakness [24, 26, 27]. Studies have shown that 4% of non-hospitalized pediatric COVID-19 patients experience long COVID symptoms within 90 days of infection [24], and children between the ages of 6 and 17 are more affected by long COVID compared to younger children [26]. Stratified research based on age suggests that children under 12 years old rarely experience long COVID symptoms, while adolescents aged 12 and above may have more pronounced symptoms but not as severe as those seen in adults [28]. The duration of long COVID symptoms is also significantly higher in adults compared to children and adolescents. This corresponds to the severity of symptoms in adults compared to children, which is consistent with the results of this study. Specifically, in the group of pediatric patients (Groups A/B) who underwent general anesthesia within 4 weeks after COVID-19 rehabilitation, a higher proportion of older children (aged 11–14) experienced transient blood oxygen desaturation during the PACU period. Therefore, for older children, especially those who may have potential long COVID symptoms, the timing of general anesthesia after COVID-19 rehabilitation should be appropriately delayed.

The present study has some limitations. Firstly, the sample size of this study is small. Secondly, the follow-up period of this study is short, and long-term complications and survival outcomes of the pediatric patients were not explored. Additionally, regarding whether the pediatric patients were infected with the Omicron variant of the novel coronavirus, this study only made speculations based on an epidemiological perspective. This study has significant implications for clinical practice in determining the timing of anesthesia for pediatric patients, providing evidence for perioperative management of pediatric patients with COVID-19, as well as offering references for the selection of surgical timing for pediatric patients after COVID-19 and other upper respiratory tract infections.

## Conclusion

Pediatric patients who have recovered from COVID-19 for more than 2 weeks have a lower risk of postoperative complications under general anesthesia. For pediatric patients with respiratory symptoms or high fever, there is a higher risk of transient decrease in blood oxygen levels during PACU period. For older pediatric patients, those with high fever, respiratory symptoms, or a longer disease course, it is recommended to appropriately extend the time from COVID-19 recovery to surgery.

## Data Availability

The datasets generated and/ or analyzed during the current study are available from the corresponding author upon reasonable request.

## Abbreviations

COVID: Corona Virus Disease
SpO_2_: Oxyhemoglobin saturation
ECG: Electrocardiograph
HR: Heart rate
BP: Blood Pressure
BIS: Bispectral index
PACU: Post-anesthesia care unit
URTI: Upper respiratory tract infections
